# HarmonyTM: multi-center data harmonization applied to distributed learning for Parkinson’s disease classification

**DOI:** 10.1117/1.JMI.11.5.054502

**Published:** 2024-09-20

**Authors:** Raissa Souza, Emma A. M. Stanley, Vedant Gulve, Jasmine Moore, Chris Kang, Richard Camicioli, Oury Monchi, Zahinoor Ismail, Matthias Wilms, Nils D. Forkert

**Affiliations:** a University of Calgary, Department of Radiology, Cumming School of Medicine, Calgary, Alberta, Canada; b University of Calgary, Hotchkiss Brain Institute, Calgary, Alberta, Canada; c University of Calgary, Biomedical Engineering Graduate Program, Calgary, Alberta, Canada; d University of Calgary, Alberta Children’s Hospital Research Institute, Calgary, Alberta, Canada; e Indian Institute of Technology, Department of Electronics and Electrical Communication Engineering, Kharagpur, West Bengal, India; f University of Alberta, Neuroscience and Mental Health Institute and Department of Medicine (Neurology), Edmonton, Alberta, Canada; g Université de Montréal, Department of Radiology, Radio-oncology and Nuclear Medicine, Montréal, Quebec, Canada; h Centre de Recherche, Institut Universitaire de Gériatrie de Montréal, Montréal, Quebec, Canada; i University of Calgary, Department of Clinical Neurosciences, Cumming School of Medicine, Calgary, Alberta, Canada; j University of Calgary, Department of Psychiatry, Calgary, Alberta, Canada; k University of Exeter, Clinical and Biomedical Sciences, Faculty of Health and Life Sciences, Exeter, United Kingdom; l University of Calgary, Department of Pediatrics, Calgary, Alberta, Canada; m University of Calgary, Department of Community Health Sciences, Calgary, Alberta, Canada

**Keywords:** shortcut learning, data harmonization, distributed learning, federated learning, traveling model

## Abstract

**Purpose:**

Distributed learning is widely used to comply with data-sharing regulations and access diverse datasets for training machine learning (ML) models. The traveling model (TM) is a distributed learning approach that sequentially trains with data from one center at a time, which is especially advantageous when dealing with limited local datasets. However, a critical concern emerges when centers utilize different scanners for data acquisition, which could potentially lead models to exploit these differences as shortcuts. Although data harmonization can mitigate this issue, current methods typically rely on large or paired datasets, which can be impractical to obtain in distributed setups.

**Approach:**

We introduced HarmonyTM, a data harmonization method tailored for the TM. HarmonyTM effectively mitigates bias in the model’s feature representation while retaining crucial disease-related information, all without requiring extensive datasets. Specifically, we employed adversarial training to “unlearn” bias from the features used in the model for classifying Parkinson’s disease (PD). We evaluated HarmonyTM using multi-center three-dimensional (3D) neuroimaging datasets from 83 centers using 23 different scanners.

**Results:**

Our results show that HarmonyTM improved PD classification accuracy from 72% to 76% and reduced (unwanted) scanner classification accuracy from 53% to 30% in the TM setup.

**Conclusion:**

HarmonyTM is a method tailored for harmonizing 3D neuroimaging data within the TM approach, aiming to minimize shortcut learning in distributed setups. This prevents the disease classifier from leveraging scanner-specific details to classify patients with or without PD—a key aspect for deploying ML models for clinical applications.

## 1 Introduction

Distributed learning has become a promising alternative to centralized learning for training machine learning (ML) models in medical image analysis, addressing many patient privacy regulations and overcoming administrative barriers.^1^ It offers a practical solution for accessing large and diverse datasets by enabling ML model training in distributed environments. Despite its success, distributed learning does not address the inherent challenge of image acquisition biases across multiple centers,^1^ which is especially important when no imaging protocol harmonization is used.

Image acquisition biases can, for example, be caused by differences in imaging acquisition protocols and/or scanner types across multiple centers, potentially leading the ML model to learn patterns unrelated to the main task (i.e., spurious correlations).^2^ Several previous studies have explored the effects of image acquisition biases. For instance, Refs. 3 and 4 demonstrated that combining data acquired with different pulse sequences or scanners in multi-center studies can lead to biases in brain volumes when conducting brain morphology analysis for cross-sectional or longitudinal studies. In addition, variations in cortical thickness measurements were observed due to distinct imaging acquisition protocols, magnetic field strength, and pulse sequence parameters.^5^^–^^7^ Moreover, Glocker et al.^8^ found that simple intensity-based harmonization techniques cannot eliminate all of a scanner’s encoded effects. Similar findings were also reported by Ref. 9, demonstrating that ML models can accurately identify the site where brain scans were acquired, even after intensity harmonization. Recently, Souza et al.^2^ showed that scanner types can be classified from the internal feature representations of a model trained for Parkinson’s disease (PD) classification in a centralized approach. Acquisition bias has also been reported for other data modalities when working with multi-center datasets such as molecular data^10^ and magnetic resonance imaging (MRI)-derived features.^11^^,^^12^ All of these studies reinforce that ML models can identify and possibly exploit image acquisition biases, such as scanner type, as potential shortcuts for the disease classification task, practically diminishing their clinical utility.^13^

Shortcuts can emerge in ML models for various reasons, as described in more detail by Geirhos et al.^13^ First, models may disproportionately focus on making good predictions for the majority group, leading to potential misclassifications in minority group(s). Second, ML models often operate with minimal effort, meaning that once a model identifies a feature to perform its task, it may rely exclusively on that feature, even if it represents a data artifact. Third, a model might learn features unrelated to the intended task, such as classifying scanner types instead of disease groups. Last, a model may combine multiple features to make a decision. For example, during a disease classification task, the model might incorrectly associate scanner type with disease status, even though the scanner used to acquire the data should not influence the diagnosis.

Hence, recent ML research has aimed to develop methods to harmonize data across different centers to reduce imaging biases and improve ML models’ reliability, generalizability, usability, and validity.^14^ Data harmonization is the process of transforming data from different sources into a common format or reference frame to enable meaningful comparisons or integration. Most data harmonization methods in ML that employ MRI volumetric data utilize generative adversarial networks (GANs).^15^^–^^20^ However, many of these methods have significant limitations, requiring paired data (e.g., data from the same individual using every scanner or image modality to be harmonized across), and large quantities of data from each site. These factors can hinder the implementation of GANs in distributed learning environments, especially in scenarios involving numerous centers, where each center contributes only a few data points for training.

Distributed learning is commonly implemented following the federated learning (FL) paradigm. In FL, all centers train a copy of a model initialized by a server in parallel. After each training round, the server aggregates the parameters from the models trained at each center, updates the global model, and sends it back to the centers for further training. This process is repeated until the aggregated model meets predefined convergence criteria.^21^ Managing centers with limited datasets presents significant challenges in FL setup, including the risk of the local models overfitting and the difficulty of defining an aggregation function that does not marginalize centers with fewer datasets.^22^ Although data harmonization methods have been proposed specifically for the FL,^23^^–^^25^ this has been limited to the scenario where centers contribute large amounts of data for training.

In contrast to FL, the traveling model (TM) visits one center at a time in a sequential process.^22^^,^^26^ Therefore, the model is initialized at a server or the first center, followed by training with the data available in the first center. Subsequently, the updated model is transferred to the next center, and training continues with locally available data. This process is repeated until the final center is reached, completing one training cycle. Multiple cycles can be conducted to enhance the model’s overall performance.^22^ Despite being less explored than FL, previous research has found that the TM approach to distributed learning is especially effective when centers are only able to provide a few datasets for training.^22^^,^^26^ Instances in which healthcare facilities possess limited data are commonly found in scenarios involving rare diseases with low prevalence,^27^ small or rural hospitals admitting a reduced number of patients, and hospitals in low- or middle-income countries due to a lack of sufficient imaging equipment, skilled clinicians, and radiologists.^28^ The effectiveness of TM arises from the iterative training of a single model, which alleviates the FL challenge of the local model’s overfitting to some extent.

Recently, the TM method has been successfully applied to classify PD using three-dimensional (3D) MRI data acquired across 83 centers worldwide.^26^ Although the TM demonstrated state-of-the-art performance on this task [achieving an area under the receiver operating characteristic curve (AUROC) of 83%, comparable with the centralized approach of 80%], it did not address or investigate the inherent challenge of image acquisition biases. In this specific example, attempting to directly apply existing data harmonization methods designed for FL to the TM approach becomes impractical as 51.8% (43 out of 83) of the centers in this PD dataset contribute less than 10 data points, with half of them (21 out of 43) contributing less than five data points for training. Unfortunately, restricted data access is a common issue in medical image analysis, which significantly affects the effectiveness of data harmonization methods, especially when insufficient data are available for training. Thus, the current state-of-the-art techniques in this domain may severely counterbalance the advantageous capability of the TM to empower centers to contribute smaller amounts of data effectively in a distributed learning scenario.

An alternative to GAN-based and FL-specific data harmonization approaches is to remove domain-specific information (e.g., scanner or acquisition protocol) from the model’s learned feature representations while trying to retain as much essential disease-related information as possible. For example, Dinsdale et al.^29^ introduced a data harmonization technique for centralized learning trained on neuroimaging data following this idea. Their work employed an adversarial training setup, with domain-specific information being “unlearned” from the features used by the model for the main task (e.g., brain age prediction). In their setup, the adversarial network comprises an encoder for extracting features from input data, a classification head for the main task, and a classification head for the domain task (i.e., scanner). Despite the effectiveness of their work, certain restrictions, such as ensuring that batches include representation from every scanner and oversampling the smallest dataset, must be adapted for a distributed environment where batches are composed of data from a single center. Adapting this method to distributed learning is challenging because centers do not share information, which limits the unlearning step, particularly in the FL setup, where training occurs in parallel. To address this challenge, Dinsdale et al.^23^ proposed an adaptation that tracks site information to generate features on the central server. Although sharing these features with each site enables the unlearning procedure to be performed effectively, it contradicts the principle of distributed learning, which aims to avoid data sharing in any way. The TM training approach not only allows centers with limited local data to participate but also eliminates the need to share information or features during the unlearning step. Thus, investigating the adversarial training setup is theoretically viable for the TM approach, although this has not yet been experimentally verified.

Therefore, this work introduces HarmonyTM, which adapts and extends the harmonization framework proposed by Ref. 29 to the TM approach, which is subsequently tested for PD classification. Overall, we aim to learn a feature representation that minimizes the effect of image acquisition biases (i.e., spurious correlations) while retaining high performance for the main task and enabling centers to contribute very small sample sizes. Our major contributions include: (1) the development of the first data harmonization method for the TM approach and (2) the evaluation of HarmonyTM in reducing the impact of scanner differences while successfully distinguishing between patients with PD and healthy participants.

## 2 Material and Methods

In this work, we evaluate HarmonyTM using the most extensive multi-center database for PD classification published to date.

### 2.1 Dataset

All analyses performed in this work utilize the unique multi-center PD database, first presented in Ref. 26, which includes 1817 T1-weighted MRI scans acquired in 83 centers worldwide.^30^^–^^42^ This database stands out for its diversity—it features a wide range of participant demographics, varying numbers of brain scans per center, multiple scanner vendors (e.g., Siemens, GE, and Phillips), and 23 different scanner types. The pre-processing of the database included skull-stripping using HD-BET,^43^ resampling to an isotropic resolution of 1 mm using linear interpolation, bias field correction using the advanced normalization tools (ANTs) non-parametric non-uniform intensity normalization technique (version 2.3.1), affine registration to the PD25-T1-MPRAGE-1mm brain atlas^44^ using ANTs, and cropping to 160×192×160 to reduce irrelevant background. Table 1 summarizes the centers’ contributions, population demographics, and scanner types.

**Table 1:** Centers’ contributions and demographics.

Centers	PD			Healthy participants			Total	Scanner type
	Size	Male (%)	Age mean (std)	Size	Male (%)	Age mean (std)		
ADNI_1	—	—	—	6	50	67.16 (1.83)	6	Siemens Prima Fit
ADNI_2	—	—	—	22	36	66.05 (4.11)	22	Siemens Prisma and GE Signa Hdxt
ADNI_3	—	—	—	2	0	64.15 (3.46)	2	GE Discovery 750
ADNI_4	—	—	—	5	40	70 (2.01)	5	Philips Ingenia
ADNI_5	—	—	—	3	66	72.06 (1.20)	3	GE Signa Hdxt
ADNI_6	—	—	—	4	50	70.75 (1.82)	4	GE Discovery 750
ADNI_8	—	—	—	6	50	63.58 (3.60)	6	Siemens Prima Fit
ADNI_10	—	—	—	2	0	60.7 (3.81)	2	Siemens Biograph mMR
ADNI_11	—	—	—	12	25	66.19 (5.34)	12	Siemens Verio
ADNI_12	—	—	—	12	41	67.59 (5.43)	12	Siemens Skyra and GE Signa Hdxt
ADNI_13	—	—	—	2	0	72.55 (2.33)	2	Philips Achieva
ADNI_15	—	—	—	6	50	67.92 (3.55)	6	Siemens Prisma and GE Discovery 750
ADNI_16	—	—	—	9	66	66.63 (4.76)	9	GE Discovery 750 and GE Signa UHD
ADNI_19	—	—	—	2	0	69 (2.12)	2	Siemens Prima Fit
ADNI_20	—	—	—	8	87	67.95 (4.01)	8	GE Discovery 750 and GE Signa Hdxt
ADNI_21	—	—	—	2	50	68.7 (7.77)	2	GE Discovery 750 and Philips Achieva
ADNI_23	—	—	—	4	50	70.4 (3.32)	4	Siemens Prisma Fit
ADNI_24	—	—	—	6	33	68.18 (2.13)	6	Siemens Skyra
ADNI_26	—	—	—	5	0	70.24 (2.37)	5	Siemens Skyra and Siemens Prisma
ADNI_27	—	—	—	4	0	67.40 (2.57)	4	Siemens Prisma
ADNI_28	—	—	—	7	28	66.94 (5.01)	7	Siemens Prisma Fit
ADNI_33	—	—	—	2	0	73.2 (1.97)	2	Siemens Prisma Fit
ADNI_34	—	—	—	7	14	61.84 (5.05)	7	Siemens Trio Tim
ADNI_37	—	—	—	6	33	68.88 (2.33)	6	GE Signa Hdxt
ADNI_38	—	—	—	8	25	69.98 (5.02)	8	GE Discovery 750 and Siemens Prisma
ADNI_39	—	—	—	2	50	70.15 (4.45)	2	GE Signa Premier
ADNI_40	—	—	—	5	40	68.42 (6.46)	5	GE Discovery 750 and Philips Achieva
ADNI_41	—	—	—	5	60	69.24 (3.14)	5	Philips Achieva
ADNI_42	—	—	—	3	33	68.13 (4.79)	3	Siemens Trio Tim
ADNI_43	—	—	—	9	44	68.8 (3.51)	9	Siemens Verio, Siemens Skyra, and Siemens Trio Tim
ADNI_44	—	—	—	9	33	68.78 (4.03)	9	Siemens Trio Tim
ADNI_47	—	—	—	12	33	69.13 (2.63)	12	GE Discovery 750
ADNI_49	—	—	—	10	20	66.11 (5.21)	10	GE Discovery 750
ADNI_50	—	—	—	8	25	68.83 (2.53)	8	Philips Achieva dStream
ADNI_51	—	—	—	2	0	66.25 (11.80)	2	Philips Ingenia
ADNI_52	—	—	—	7	71	67.78 (2.01)	7	GE Discovery 750
ADNI_54	—	—	—	2	50	68.65 (1.06)	2	Siemens Skyra
ADNI_55	—	—	—	12	25	67.67 (3.69)	12	Siemens Skyra and Siemens Verio
ADNI_58	—	—	—	22	27	67.67 (3.75)	22	Siemens Prisma Fit
ADNI_59	—	—	—	3	0	69.53 (1.20)	3	Siemens Prisma Fit and Philips Achieva
ADNI_60	—	—	—	8	37	67.6 (5.82)	8	GE Discovery 750 and Philips Ingenia
ADNI_61	—	—	—	2	0	72.6 (2.54)	2	Siemens Trio Tim and Philips Ingenia
BIOCOG	45	55	71.1 (4.48)	49	57	71.47 (4.85)	94	Siemens Sonata
C-BIG	66	54	65 (8.43)	10	10	62.4 (11.88)	76	Siemens Prisma Fit
HAMBURG	74	70	63.62 (8.95)	39	61	62.30 (11.71)	113	Siemens Skyra
HMC	3	33	71.93 (5.81)	—	—	—	3	GE Discovery 750
Japan	30	43	67.56 (6.80)	15	46	63.33 (5.24)	45	Siemens Verio
JGH	2	100	71.4 (11.87)	—	—	—	2	Siemens Trio Tim
MUC	10	70	65.66 (7.71)	—	—	—	10	Siemens Trio Tim and Siemens Prisma Fit
Neurocon	26	61	68.76 (10.75)	16	25	67.62 (11.88)	42	Siemens Avanto
OASIS	—	—	—	27	62	66.33 (7.17)	27	Siemens Trio Tim and Siemens Biograph mMR
CALGARY	79	67	71.31 (6.41)	42	47	69.8 (6.87)	121	GE Discovery 750
PLS	41	63	61.81 (5.75)	21	47	62.85 (6.36)	62	Siemens Trio Tim
PPMI_10	16	56	63.21 (7.93)	7	57	63.42 (16.70)	23	GE Discovery 750 and GE Signa Hdxt
PPMI_12	19	52	66.42 (9.14)	10	50	58.6 (13.87)	29	Philips Achieva
PPMI_13	22	77	58.31 (9.16)	5	40	66 (4.74)	27	Siemens Trio Tim
PPMI_14	2	100	65.5 (9.19)	—	—	—	2	Siemens Trio Tim
PPMI_15	16	68	62.43 (13.44)	10	50	60.3 (12.12)	26	GE Optima MR450 and GE Signa Hdxt
PPMI_16	19	63	58.21 (9.49)	8	50	61.37 (9.05)	27	GE Signa Hdxt and Philips Gyroscan NT
PPMI_17	11	72	59.18 (12.48)	9	66	64 (13.32)	20	GE Signa Hdxt
PPMI_18	13	53	66.3 (7.27)	4	100	62 (10.23)	17	Siemens Trio Tim
PPMI_19	22	68	59.45 (10.30)	12	66	52.75 (13.92)	34	Siemens Trio Tim and Siemens Espree
PPMI_20	27	74	60.77 (10.27)	13	46	58.76 (10.29)	63	GE Genesis Signa, GE Signa Excite, Siemens Espree, and Siemens Symphony
PPMI_21	14	78	57.92 (7.94)	—	—	—	14	Philips Gyroscan NT and Philips Intera
PPMI_22	18	77	62.61 (7.93)	12	83	61.91 (12.41)	30	Philips Achieva and GE Signa Hdxt
PPMI_23	12	25	62 (8.71)	12	75	64 (8.89)	24	Siemens Trio Tim and Siemens Espree
PPMI_25	19	73	59.31 (9.36)	9	77	56.55 (12.81)	28	Siemens Trio Tim
PPMI_26	14	57	62.14 (9.34)	1	100	63 (0.00)	15	GE Genesis Signa, GE Signa Hdxt, and Siemens Espree
PPMI_27	21	57	61.38 (9.03)	11	72	56.45 (10.80)	32	Siemens Trio Tim and GE Signa Hdxt
PPMI_28	20	65	61.29 (10.44)	5	80	58.2 (10.91)	25	Siemens Trio Tim
PPMI_29	11	36	66 (10.17)	6	100	67.83 (10.45)	17	Siemens Trio Tim, Siemens Espree, and Siemens Symphony
PPMI_30	3	66	62 (5.56)	2	100	71.5 (13.43)	5	Siemens Verio
PPMI_51	18	61	63.27 (7.75)	7	71	58.85 (6.91)	25	Siemens Trio Tim
PPMI_52	23	65	64.22 (9.54)	11	36	64.09 (6.65)	34	Siemens Trio Tim
PPMI_53	5	40	53.4 (13.64)	7	42	49.85 (14.12)	12	Siemens Verio
PPMI_55	3	100	61 (13.22)	1	100	67 (0.00)	4	Siemens Verio
PPMI_59	6	83	60 (12.71)	—	—	—	6	Philips Intera
RUH	6	50	71.05 (1.90)	—	—	—	6	Siemens Skyra
SALD	—	—	—	78	100	61.65 (8.03)	78	Siemens Trio
SBK	3	100	73.5 (3.97)	—	—	—	3	Siemens Prisma
Taowu	17	47	64.52 (4.19)	20	60	64.75 (5.58)	37	Siemens Trio
UKBB	48	58	70.02 (5.91)	197	60	66.2 (7.76)	245	Siemens Skyra
UOA	33	63	67.7 (8.02)	—	—	—	33	Siemens Prisma

### 2.2 Travelling Model

In this work, we implemented the PD TM originally presented in Ref. 26 as the basis for all experiments. In essence, this approach involves defining an initial traveling sequence that determines the order in which the model is transferred between centers. Following this, the first center initializes and trains the model with the locally available data before passing it on to the next center. This training process continues until the model has visited every center, completing one cycle. Subsequently, a new traveling sequence is defined to introduce cycle-to-cycle variability, effectively simulating the batch shuffling process commonly used in centralized approaches. A batch size of five is employed when a center has five or more locally available datasets. In cases where fewer than five datasets are available, the batch size is adjusted accordingly. This variation was needed because 21 of the 83 centers in the distributed learning network had fewer than five datasets available for local training. In addition, computational limitations restrict the maximum batch size to five at any given time. The Adam optimizer began with an initial learning rate of 0.0001 and employed exponential decay throughout each cycle as described in Ref. 26. Moreover, the entire training process is iterated for 30 cycles, with only one epoch of training occurring at each center to improve the model’s performance. Although the training was conducted on a single computer equipped with an NVIDIA GeForce RTX 3090 GPU, it strictly adheres to the TM concept by retrieving data from one center per epoch. The entire training process takes ∼1.5  h to complete. Details of the deep learning architectures used in this work are described in Sec. 2.3.

### 2.3 Harmonization Strategy

The deep learning architecture used in this work is based on the state-of-the-art simple fully convolutional network (SFCN),^45^ which achieved high performance for PD classification using multi-center T1-weighted MRI scans in centralized and TM approaches.^26^^,^^46^ The model’s encoder comprises seven blocks: The initial six blocks mirror the structure of the original SFCN model. These include five blocks featuring a 3D convolutional layer with 3×3×3 kernel filters, batch normalization, 2×2×2 max pooling, and ReLU activation, whereas one block incorporates a 3D convolutional layer with 1×1×1 kernel filters, batch normalization, and ReLU activation. The last block is tailored for our specific task and includes a 3D average pooling layer, a dropout layer with a 0.2 rate, and a flattening layer with 768 features (neurons). The classification head utilizes the encoder output and consists of a single dense layer using a sigmoid activation function for the binary output, distinguishing between patients with PD and healthy participants. Meanwhile, the domain head is a single layer employing a softmax activation function for the multiclass output, categorizing 23 different scanner types.

**Fig. 1:**
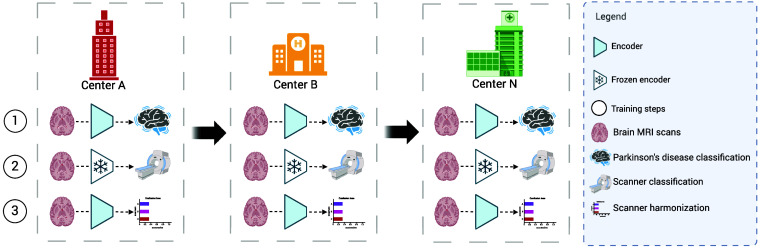
HarmonyTM steps to remove scanner-specific information from the feature representation of the PD classification model.

Before removing domain-specific details, it is necessary to pre-train the network components with the TM approach. Therefore, the encoder and disease classification head are initially trained until convergence. Following this, the encoder is frozen, and the scanner classification head is trained until convergence. Finally, utilizing these pre-trained models, the scanner harmonization procedure is implemented in three steps as follows. It is important to highlight that these steps are performed for each batch, such that the three training steps occur at each center before transferring the model to the next center (see Fig. 1).

1. Optimize the encoder and disease classification head for the PD classification task.
2. Optimize the scanner classification head for identifying scanners from the feature representation of the frozen encoder that is trained in step 1.
3. Optimize the encoder by employing an adversarial confusion loss to eliminate scanner-specific information. This loss guides the scanner classification head output toward chance-level performance. In essence, chance-level means that the model would make predictions purely by random guessing, eliminating any shortcuts related to scanners that might be exploited for disease classification.

The scanner harmonization process involves the application of four distinct losses. In the first step, a PD classification loss [Eq. (1)] is utilized to minimize the binary cross entropy. This consists of adjusting the model’s parameters to diminish the difference between its predictions and the ground truth labels (PD versus HP). In the second step, a scanner classification loss [Eq. (2)] is employed with a similar objective as in step 1. However, categorical cross-entropy is minimized in this instance, given that the prediction involves identifying scanner types and only the domain head is optimized in this step. An adversarial confusion loss [Eq. (3)] is computed in the third step, introducing a counteractive objective to step 2. Step 2 strives for the model to recognize scanner types precisely, whereas step 3 aims to counteract this ability by removing information related to scanner types from the feature representations, where N represents the batch size, which varies depending on the amount of data available at each center. In the end, the total loss [Eq. (4)] is computed as the sum of the three losses described. This cumulative loss is subsequently used to optimize the encoder. Compared with the implementation proposed in Ref. 29, two important adaptations were made. (1) Introducing N to manage varying batch sizes during training. In our case, N accounts for the variation in batch size due to differing amounts of data across centers. (2) Eliminating the requirement for oversampling, which was used in the original method^29^ to ensure that every scanner type was represented in each batch. Their centralized approach allowed for control over such a batch composition, ensuring the representation of every scanner type and oversampling underrepresented types to achieve balance. However, this is not feasible in the TM approach, where each center only has access to its own data. The harmonization process was iterated through 30 cycles, taking ∼2.5  h to complete. The code is available in a GitHub repository available at: https://github.com/RaissaSouza/scanner-harmonization:

Lpd(y,y^)=−1N∑n=1Nyn×log(y−y^), (1)

Lsc(y,y^)=−1N∑i=1N∑j=1Myi,j×log(y^i,j), (2)

Lconf(y^)=−∑n=1N1N×23 log(y^n), (3)

Ltotal=Lpd+Lsc+Lconf. (4)

### 2.4 Baseline

As a baseline for comparison to our TM approach, we trained an identical deep learning architecture as outlined in Sec. 2.3 in a centralized fashion. Here, both the model and the entire database are accessible at a single center, where training takes place. We utilize a batch size of five with shuffling and an initial learning rate of 0.001 to keep the results comparable as much as possible. Unlike the TM approach, where each batch only includes data from the same center, in the centralized approach, batches may comprise data from various centers. The two adaptations made for the TM approach—incorporating N and eliminating oversampling—were maintained in the centralized approach.

### 2.5 Task Evaluation

To quantitatively evaluate the performance of the PD classification, we employed the following key metrics: the AUROC, accuracy, sensitivity, specificity, precision, and F1-score. AUROC measures the model’s ability to distinguish between patients with PD and HP across all possible logit thresholds. In contrast, classification accuracy measures the overall correctness of the model by calculating the ratio of correctly predicted instances to the total instances for a fixed threshold of 0.5. Sensitivity measures the proportion of patients with PD correctly identified by the model, whereas specificity measures the proportion of healthy participants correctly identified by the model. Precision assesses how many predicted positives are actually true positives. Finally, the F1-score, which is the harmonic mean of precision and sensitivity, provides a balanced measure that accounts for both false positives and false negatives. In all cases, a higher score indicates a better predictive performance.

The accuracy, sensitivity, specificity, precision, and F1-score metrics were also employed to assess the scanner information within the trained model’s feature representation. As the scanner classification is a multi-class problem, the weighted average accounting for class size was employed for each metric. In addition, confusion matrices were employed to visually investigate the degree of information encoded for each scanner type.

### 2.6 Feature Representation Evaluation

We utilized an unsupervised ML approach to evaluate how the proposed harmonization procedure affects the encoding of information related to scanner type and disease status (PD versus HP) within the model. This technique employs principal component analysis (PCA) to investigate the underlying patterns within the features of the last encoder’s layer, as proposed in Ref. 47. Following this step, similar to Ref. 47, we generated two-dimensional scatter plots for the first two dominant PCA modes, considering both the scanner type and the presence of disease. Finally, logistic regression was employed to measure the degree to which scanner types and main task classes (PD versus HP) can be linearly separated within each PCA mode.

## 3 Results

**Fig. 2:**
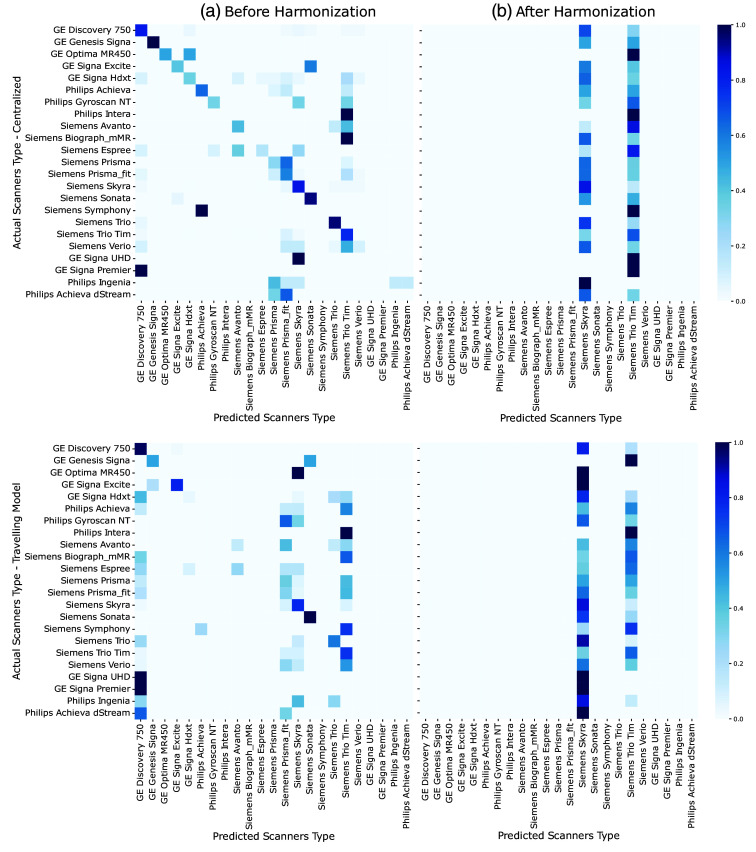
Confusion matrices of the scanner predictions for the centralized and TM approaches. Panel (a) illustrates classification before harmonization, whereas panel (b) presents them after harmonization.

The results of this work show that the PD classification performance of the TM not only remains stable but also improves after removing scanner-specific information from the feature representation (see Table 2). As expected, the scanner classification performance drops across all metrics after harmonization. Moreover, it can be observed that the TM approach is less prone to encoding scanner information (53% accuracy) before harmonization when compared with the centralized approach (65% accuracy). Nevertheless, in both cases, the harmonization method reduces scanner classification abilities to 30% accuracy and sensitivity, 96% specificity, 12% precision, and 16% F1-score. Figure 2 supports these findings as illustrated by the confusion matrices of the centralized and TM approaches before and after harmonization. The matrices display the counts of predictions for each pair of actual and predicted classes, where each row represents the actual scanner types, and each column represents the predicted scanner types. In Fig. 2(b), it is evident that after scanner harmonization, the majority of datasets are mistakenly classified as Siemens Skyra and Siemens Trio Tim. Together, these two scanners constitute the largest portion (37.5%) of the test set (Fig. S1 in the Supplementary Material shows the proportion of each scanner type in the test set). Furthermore, our results demonstrate that the TM approach leads to the greatest improvements in PD classification after scanner harmonization, achieving 76% accuracy, 82% AUROC, 83% sensitivity, and 75% F1-score compared with the centralized approach. None of the models showed improvements in specificity and precision after harmonization, although their performance remained comparable.

**Table 2:** Parkinson’s disease and scanner classification performances before and after scanner harmonization.

Training approach	Scanner harmonization	Accuracy	AUROC	Sensitivity	Specificity	Precision	F1-score
PD classification performance							
Centralized	Before	0.75	0.79	0.69	0.80	0.74	0.71
	After	0.74	0.81	0.75	0.73	0.70	0.73
TM	Before	0.72	0.78	0.67	0.75	0.70	0.68
	After	0.76	0.82	0.83	0.69	0.69	0.75
Scanner classification performance							
Centralized	Before	0.65	N/A	0.69	0.98	0.63	0.62
	After	0.30		0.30	0.96	0.12	0.16
TM	Before	0.54		0.54	0.98	0.43	0.46
	After	0.30		0.30	0.96	0.12	0.16

**Fig. 3:**
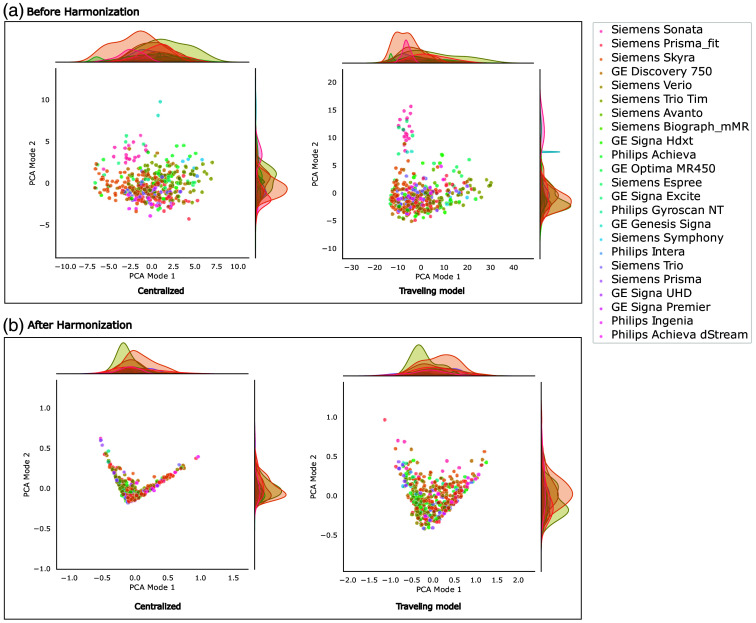
2D scatter plots of the first two PCA modes, considering the scanner type. Panel (a) illustrates the scatterplots before harmonization, whereas panel (b) presents the scatterplots after harmonization.

**Fig. 4:**
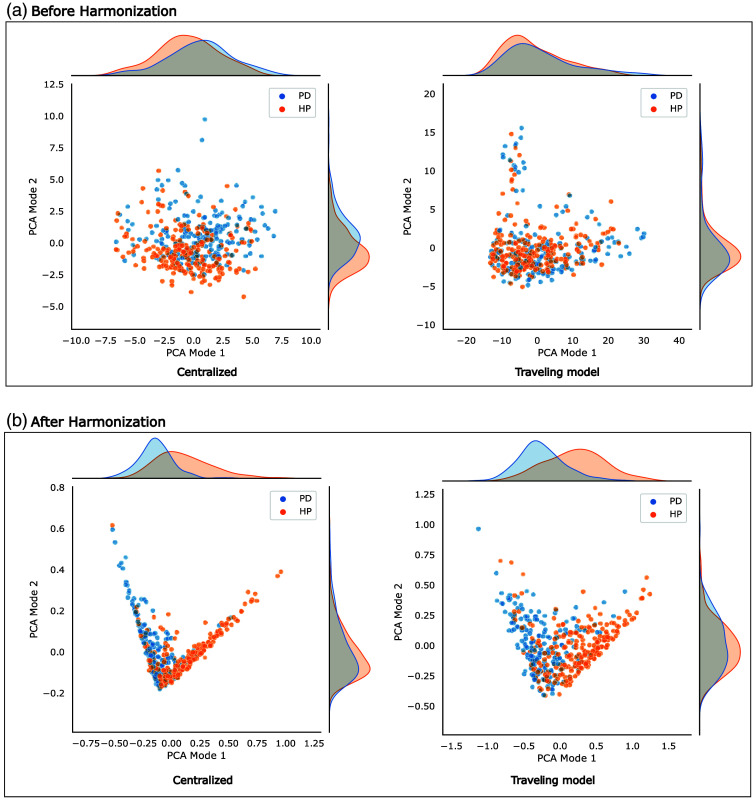
2D scatter plots of the first two PCA modes, considering the presence of disease. Panel (a) illustrates the scatterplots before harmonization, whereas panel (b) presents the scatterplots after harmonization.

Figures 3 and 4 display the distributions of the first two modes of PCA applied to the feature representations of the models, with color-coded by scanner type in Fig. 3 and color-coded by disease class in Fig. 4. In Fig. 3, it can be seen that before scanner harmonization, the distribution of scanner types within the feature representation is more distinct and less overlapping compared with the corresponding results after harmonization. In contrast, Fig. 4 shows that the distributions of patients with PD and healthy participants are more similar before harmonization but become more distinguishable after harmonization. Logistic regression analysis reveals that, before scanner harmonization, PCA mode 1 encodes information for disease classification, achieving accuracies of 58% and 53% for the centralized and TM approaches, respectively. After harmonization, these percentages increase to 71% and 75%, respectively. Moreover, logistic regression analysis revealed that some information is encoded through PCA modes 1 and 2 for scanner classification, yielding accuracies of 38% and 35% before harmonization, which subsequently decreases to 28% after harmonization. The complete logistic regression analysis results are provided in Tables S1 and S2 in the Supplementary Material.

## 4 Discussion

In this work, we introduced HarmonyTM, the first data harmonization method specifically designed and evaluated for the TM approach. The results showed that HarmonyTM is effective in creating a feature representation that reduces image acquisition biases (i.e., spurious correlations) and enhances disease-related information, achieving the highest classification performance for PD after scanner harmonization. We specifically focused this first feasibility analysis on scanners because of the well-known bias effects.^9^ Although our study demonstrated the effectiveness of HarmonyTM in harmonizing imaging data from different scanners using T1-weighted MRI scans, the method can be readily adapted to address other potential spurious correlations and different imaging modalities. However, further experimental validation is needed to confirm its broader applicability.

It is crucial to highlight that the TM approach itself was found less prone to learning spurious correlations than the centralized approach even before data harmonization. This resistance may be attributed to the fact that each training data batch originates from a single center in the TM setup. By avoiding the inclusion of data from multiple centers in a single batch, the likelihood of the model learning image acquisition biases associated with different factors (e.g., scanner types) across centers is reduced. Conversely, the TM approach’s use of single-center batches may explain why it achieved less balanced metrics compared with the centralized approach after harmonization. The centralized approach, which includes data from multiple centers in each batch, increases the likelihood of training in batches where both classes are represented. This contrasts with the TM approach, where training batches from centers such as OASIS, SALD, and ADNI consist only of healthy participants, and centers such as UOA, RUH, and some from PPMI include only PD cases.

Our results indicate that before scanner harmonization, the difference in the distribution of patients with PD and healthy participants within the feature representation of the last encoder’s layer was poorly noticeable in the centralized as well as TM approach. Conversely, the distributions of scanner types exhibited more noticeable differences, allowing for some degree of classification, with a higher level of accuracy of the logistic regression model from the two PCA modes computed from the model trained using the centralized approach compared with the model trained using the TM approach. These observations are consistent with previous research,^2^ which demonstrated that the feature representation of a PD classifier trained with the centralized approach can be used directly for classifying scanner types, achieving even better performance in scanner type classification compared with PD classification. Following scanner harmonization, a shift occurred: the distinctions between patients with PD and healthy participants became more pronounced, whereas the differences in scanner-type distributions became less prominent. This pattern aligns with the results reported by Dinsdale et al.,^29^ who initially introduced this method for the centralized training approach. Although their research implies that removing image acquisition biases could lead to a loss of disease-related information when the entire dataset is used for harmonization, we chose to apply harmonization to the full training dataset. As a result, we did not impose restrictions based on data availability, which could substantially reduce center participation in real-life scenarios. Despite these concerns, we found that HarmonyTM benefits from using the full dataset for harmonization. Rather than negatively impacting performance, this approach led to improved disease classification outcomes.

It is essential to highlight some of the limitations of this work. First, we utilized a single established PD classifier model, which was trained using a realistic multi-site database. However, only one specific but widely used image modality (T1-weighted MRI) was investigated in this work for harmonization. Therefore, the good performance of HarmonyTM in other scenarios involving different deep learning architectures, other medical imaging modalities, and additional medical imaging tasks has yet to be demonstrated. It is crucial to recognize that biases must be first identified in the data, as it is impossible to unlearn unknown biases. It is noteworthy, however, that our work utilized the largest PD multi-center database, encompassing various scanner types and small datasets from many centers, compared with the datasets used in many other multi-center analyses, potentially enhancing the generalizability of results. Moreover, our investigation was limited to a single neuroimaging modality—T1-weighted MRI data. Although HarmonyTM can be applied to any two-dimensional (2D) or 3D data, the results may vary depending on the degree of spurious correlations present in the specific image modality. Furthermore, although we used a computer equipped with an NVIDIA GeForce RTX 3090 GPU, we believe that any center with comparable resources should be capable of training HarmonyTM. Last, it is important to note that we used only one training epoch at each center per cycle to minimize the risk of overfitting. However, we did not assess the performance at each center to ensure that overfitting did not occur. Nevertheless, additional strategies, such as regularization and the use of an external validation set, could be implemented to further analyze and prevent overfitting. Future work could address several limitations identified in this study. These include exploring HarmonyTM’s effectiveness across different deep learning architectures, medical imaging modalities, and diagnostic tasks to assess its generalizability. In addition, further investigation into strategies for mitigating overfitting risks and determining the minimal computational resources required would enhance its viability for under-resourced centers.

## 5 Conclusion

This work introduced HarmonyTM, a method specifically designed for harmonizing 3D MRI data in the context of the TM approach. HarmonyTM tackles the issue of image acquisition biases across different centers, a common challenge in systems that learn from data distributed across multiple locations. To the best of our knowledge, this is the first work implementing a data harmonization method for the TM approach. Our findings demonstrate the effectiveness of HarmonyTM in generating features with reduced influence from image acquisition biases, such as scanner types, while not only maintaining but also improving performance in classifying PD. In addition, our results emphasize that the TM approach has inherent resistance to learning image acquisition biases. This aspect is crucial for developing clinically useful deep learning models with broad applicability.

## Supplementary Material



## Data Availability

Image data used were provided in part by the UK Biobank (application number 77508), by the PPMI-a, a public–private partnership funded by the Michael J. Fox Foundation by the OASIS-3 project (principal investigators: T. Benzinger, D. Marcus, J. Morris; NIH P50 AG00561, P30 NS09857781, P01 AG026276, P01 AG003991, R01 AG043434, UL1 TR000448, R01 EB009352), by the OpenfMRI database (accession number ds000245), and by the Alzheimer’s Disease Neuroimaging Initiative (ADNI), a partnership involving multiple centers across North America with the goal of tracking participants through periods of cognitive decline and dementia. Launched in 2003, ADNI continues to evaluate biomarker, neuroimaging, and neuropsychological status in participants.
