# Supplementary material for: HarmonyTM: multi-center data harmonization applied to distributed learning for Parkinson’s disease classification
Source: J Med Imaging (Bellingham). 2024 Sep 20;11(5):054502. doi: 10.1117/1.JMI.11.5.054502 (PMC11413651; doi:10.1117/1.JMI.11.5.054502)
Supplement: Supplementary file 1 [file JMI_011_054502_SD001.pdf]

Supplementary material

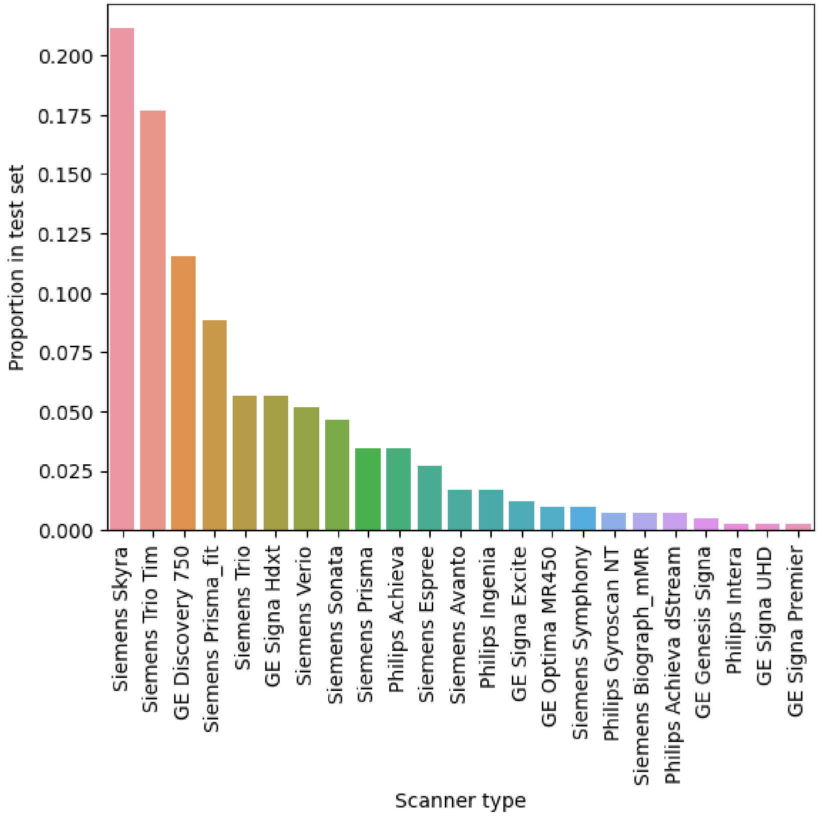

Figure 1: Proportions of samples from each scanner type in our test set.

Table 1: Logistic regression analysis **before** scanner harmonization. CL = centralized learning and TM = travelling model

**Modes**	**CL - disease**	**TM - disease**	**CL - scanner**	**TM - scanner**
1 & 2	0.665	0.538	0.385	0.351
1	0.584	0.533	0.292	0.287
2	0.636	0.545	0.314	0.260

Table 2: Logistic regression analysis **after** scanner harmonization. CL = centralized learning and TM = travelling model

**Modes**	**CL - disease**	**TM - disease**	**CL - scanner**	**TM - scanner**
1 & 2	0.714	0.739	0.289	0.285
1	0.719	0.751	0.287	0.272
2	0.562	0.540	0.213	0.265
